# Antioxidant mesoporous Ce-doped bioactive glass nanoparticles with anti-inflammatory and pro-osteogenic activities

**DOI:** 10.1016/j.mtbio.2020.100041

**Published:** 2020-01-09

**Authors:** Kai Zheng, Elisa Torre, Alessandra Bari, Nicola Taccardi, Clara Cassinelli, Marco Morra, Sonia Fiorilli, Chiara Vitale-Brovarone, Giorgio Iviglia, Aldo R. Boccaccini

**Affiliations:** a Institute of Biomaterials, University of Erlangen-Nuremberg, Erlangen, Germany; b Nobil Bio Ricerche Srl, Portacomaro D'Asti, Italy; c Department of Applied Science and Technology, Politecnico di Torino, Turin, Italy; d Institute of Chemical Reaction Engineering, University of Erlangen-Nuremberg, Erlangen, Germany

**Keywords:** Bioactive particles, Cerium, Surface modification, Anti-inflammatory, Antioxidant, Pro-osteogenesis

## Abstract

Mesoporous bioactive glass nanoparticles (MBGNs) are emerging biomaterials for bone repair/regeneration, considering their favorable pro-osteogenic and proangiogenic activities. To further improve their therapeutic effects, the endowment of MBGNs with additional antioxidant properties is of particular interest to target oxidative stress related to bone remodeling and diseases. To this end, we developed antioxidant cerium-containing MBGNs (Ce-MBGNs) (particle size of 100–300 ​nm) by using a postimpregnation strategy to incorporate Ce, through which the shape, pore structure, and dispersity of the nanoparticles were preserved. The incorporated amount of Ce could be tailored by adjusting the concentration of the Ce precursor solution. When impregnated at a relatively low temperature (20 ​°C), Ce-MBGNs containing either 1.8 or 2.8 ​mol% of Ce were produced, while the formation of by-product cerium oxide nanoparticles (nanoceria) could be avoided. In both developed Ce-MBGNs, the concentration of Ce^4+^ was higher than that of Ce^3+^, while the relative molar percentage of Ce^4+^ was similar (∼74%) in both Ce-MBGNs. The obtained Ce-MBGNs were evidenced to be non-cytotoxic against fibroblasts at the concentration of 1 ​mg/mL. Moreover, the incorporation of Ce into MBGNs significantly reduced the expression of oxidative stress–related genes in macrophages (J774a.1). Particularly in the presence of pro-oxidation agents, Ce-MBGNs could downregulate the expression of oxidative stress–related genes in comparsion with the polystyrene plates (control). When cultured with Ce-MBGNs, the expression of proinflammatory-related genes in macrophages could also be downregulated in comparsion with MBGNs and the control. Ce-MBGNs also exhibited pro-osteogenic activities through suppressing pro-osteoclastogenic responses. The obtained results highlight the great potential of the developed Ce-MBGNs in a variety of biomedical applications, particularly in treating bone defects under inflammatory conditions, considering their antioxidant, anti-inflammatory, and pro-osteogenesis activities.

## Graphical abstract

**Figure:**
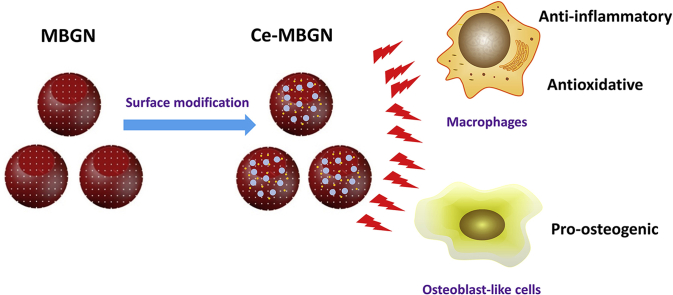

## 1 Introduction

Bioactive glasses (BGs) are versatile multifunctional biomaterials, suitable for numerous biomedical applications, for example, from bone regeneration and wound healing to cancer treatment [1,2]. Depending on their chemical composition and morphology, BGs can induce osteogenesis, angiogenesis, or antibacterial action to different extents [3]. Their morphology can be also tailored for specific applications [1,4]. For instance, spherical bioactive glass nanoparticles (BGNs) are preferred as drug delivery carriers or bioactive fillers in injectable biomaterials because of their unique flow properties [5,6]. Moreover, mesoporous BGNs (MBGNs), given their large specific surface area (SSA) and tunable pore structure (e.g., pore volume, pore size) [7,8], are particularly attractive carriers for the codelivery of drugs (e.g., antibiotics, growth factors) [9] and biologically active ions (e.g., Cu ions) [8]. Such a codelivery of ions and biomolecules has been reported to be able to induce synergistic effects toward ​enhanced therapeutic outcomes (e.g., osteogenesis, angiogenesis) [10,11]. MBGNs are thus attracting increasing attention in tissue regeneration and nanomedicine, given their desired morphological and compositional characteristics [8–12].

Oxidative stress, induced by reactive oxygen species (ROS), can cause adverse effects on cells and tissues, for example, inducing specific oxidation of some enzymes or protein degradation [13]. Reduction of excessive ROS is thus necessary to retain healthy biological functions, which *in vivo* is usually achieved by enzymatic antioxidants (e.g., superoxide dismutase, catalase) [14]. However, an imbalance of *in vivo* redox homeostasis in favor of ROS can disrupt redox signaling/control and cause oxidative stress, which can damage DNA, proteins, or cells and eventually induce inflammatory and pathological responses [[13], [14], [15]]. On the other hand, active modulation of microenvironment ROS can lead to regulated inflammatory responses, which further promotes desired biological activities, for example, enhanced osteogenesis/angiogenesis, for bone regeneration [[14], [15], [16], [17]]. In this context, for example, titanium implants functionalized with antioxidant cerium oxide coating have shown significantly promoted bone formation and osseointegration, due to actively reduced ROS levels [18].

Endowing biomaterials with antioxidant activity can reduce *in vivo* inflammatory responses [19] and promote osteogenesis and angiogenesis [18,20]. On the other hand, conventional BG compositions (e.g., 45S5 BG composition) have negligible antioxidant activity [21,22], and thus many efforts have been dedicated to enhance the antioxidant property. To this aim, surface functionalization with antioxidant species such as natural polyphenols has been reported to greatly improve the antioxidant activity of BGs without significantly affecting their intrinsic bioactivity and biocompatibility [21]. Alternatively, incorporation of elements with well-known antioxidant behavior (e.g., cerium, selenium) into BGs can also enhance their antioxidant activity [23,24]. Particularly, cerium (Ce) can switch in the oxidation states between Ce^4+^ and Ce^3+^ during redox reactions in physiological fluids [25]. Such a switch can quench free radicals including ROS and tune the oxygen situation within the microenvironment, consequently inducing anti-inflammatory, pro-osteogenesis, and proangiogenesis activities [18,[26], [27], [28]]. For example, the incorporation of cerium oxide nanoparticles (nanoceria) into 70S BG (70SiO_2_–30CaO, in mol%) scaffolds was proved to enhance the osteoblastic differentiation and collagen production of human mesenchymal stem cells [29]. In addition to 3D scaffolds, hybrid particles composed of 45S5 BG and nanoceria have been synthesized using a flame synthesis technique, whose size and Ce^3+^/Ce^4+^ ratio could be tailored by tuning processing parameters (e.g., the concentration of Ce precursor) to achieve specific properties such as catalytic and antibacterial activities [30]. Besides crystalline nanoceria, Ce can also be incorporated into the BG framework to produce chemically homogenous Ce-containing BGs (Ce-BGs) [23,29,[31], [32], [33], [34]]. Particularly, these Ce-BGs can preserve the morphology and microstructure of the BG matrix (e.g., porosity, nanoparticle dispersity) because of the absence of nanoceria clusters [35,36]. To this purpose, Ce-containing non-porous BGNs exhibiting both Ce^3+^ and Ce^4+^ oxidation states have been synthesized using a sol-gel–based approach [37]. Mesoporous BG powders can also be synthesized using sol-gel–based strategies [35,36]. However, to the best of our knowledge, development of Ce-containing MBGNs (Ce-MBGNs) has not been reported so far, although these nanoparticles are of great interest to the applications as bioactive fillers and drug delivery platforms.

In this study, we aimed to develop Ce-MBGNs with improved chemical homogeneity and dispersity, which could act as drug delivery carriers or bioactive fillers for bone repair/regeneration applications. To this end, we selected a two-step approach, according to which MBGNs (SiO_2_–CaO composition) were first prepared using an established template-assisted sol-gel method [38] followed by the incorporation of Ce through a postimpregnation process of the preformed particles. The proposed strategy could avoid the potential risk of particle agglomeration and formation of by-products (e.g., nanoceria) because of the direct addition of metallic precursors during sol-gel processes [37,39]. The synthesized Ce-MBGNs were comprehensively characterized in terms of morphology, microstructure, composition, Ce oxidization states, and dissolution behavior. We further evaluated the effects of Ce incorporation on various *in vitro* biological responses of cells, including oxidative stress, inflammatory response, and osteogenesis.

## 2 Materials and methods

### 2.1 Materials

Cetrimonium bromide (CTAB), ethyl acetate (EA), aqueous ammonia (1M), tetraethyl orthosilicate (TEOS), calcium nitrate tetrahydrate (CaN), ethanol (96%), Cerium(III) nitrate hexahydrate, potassium bromide (KBr), nitric acid (HNO_3_), hydrochloric acid (HCl, 37%), hydrofluoric acid (HF), and sodium nitroprusside (NPS) were purchased from Sigma-Aldrich (Darmstadt, Germany) without further purification. Ultrapure water was obtained with Milli-Q equipment (Millipore, Billerica, MA, USA). Dulbecco's phosphate-buffered saline (DPBS, pH ∼7.4) and Dulbecco's modified Eagle's medium (DMEM) were obtained from Gibco Invitrogen (Cergy-Pontoise, France). Fetal bovine serum (FBS), streptomycin, penicillin, l-glutamine, 3-(4,5-dimethylthiazol-2-yl)-2,5-diphenyltetrazolium bromide (MTT), and dimethylsulphoxide were purchased from Thermo Fisher Scientific (Waltham, USA). Minimum Eagle's Medium (MEM) was purchased from BIOCHROM KG (Berlin, Germany). Trypsin-Ethylenediaminetetraacetic acid (EDTA) solution was obtained from Life Technology (Carlsbad, USA).

### 2.2 Synthesis of MBGNs

MBGNs were synthesized using a microemulsion-assisted sol-gel method, as reported in the literature [40]. Briefly, 0.7 ​g of CTAB was first dissolved in 33 ​mL of deionized water under continuous stirring before the addition of 10 ​mL of EA. After stirring for 30 ​min, 7 ​mL of aqueous ammonia (1M) was added with a further 15 ​min of stirring. TEOS (3.6 ​mL) was then added with ​30 ​min of stirring, and subsequently, 2.28 ​g of CaN was added. The resulting mixture was then stirred for an additional 4 ​h. The formed nanoparticles were collected by centrifugation and washed twice with deionized water and once with ethanol. The collected particles were then dried at 60 ​°C overnight before calcination in air at 700 °​C for 3 ​h with a heating rate of 2 ​°C/min in a furnace (L 5/11, Nabertherm, Germany).

### 2.3 Postmodification of MBGN

**Fig. 1:**
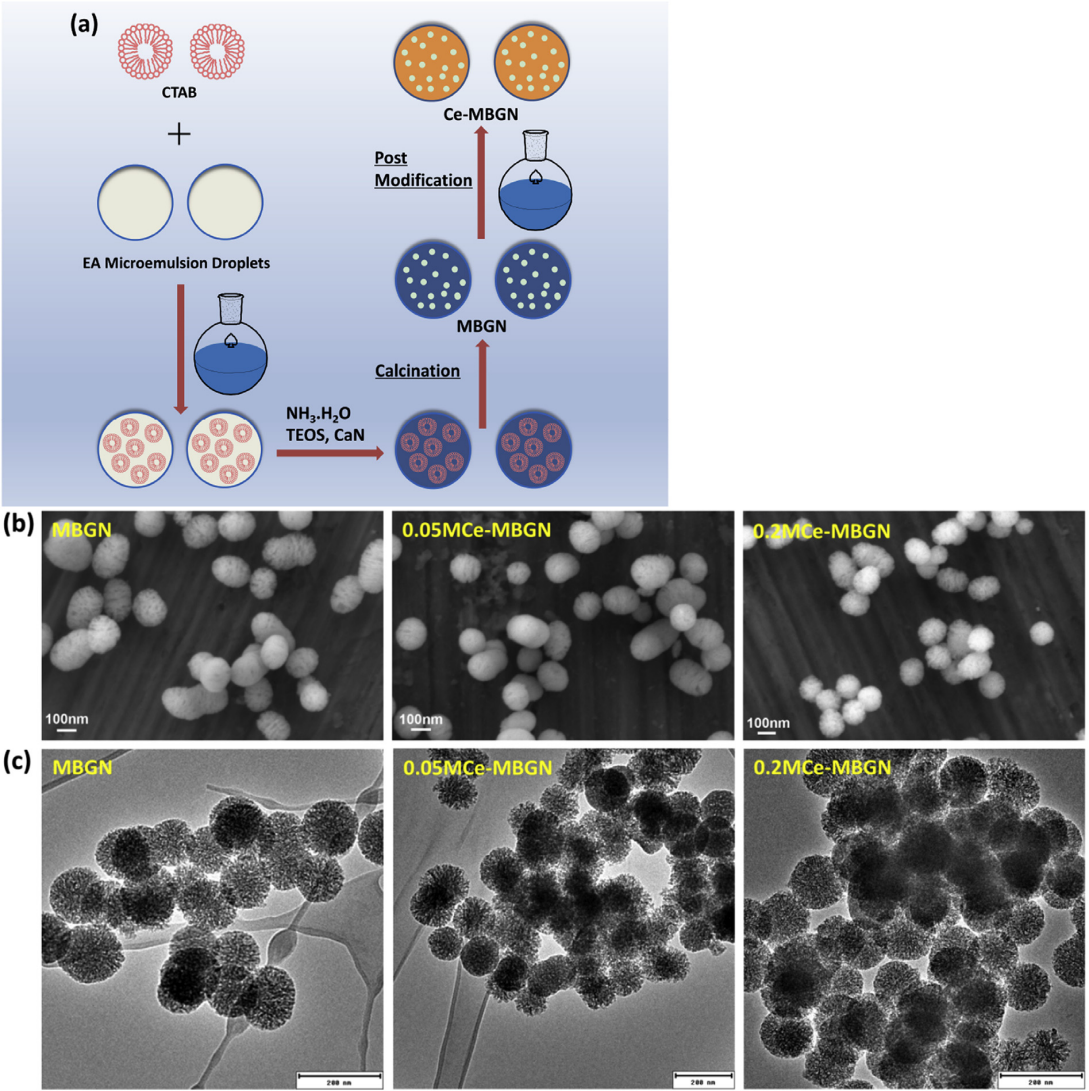
(a) Schematic illustration of MBGN synthesis and the postmodification process. (b) SEM images of the morphology of MBGN, 0.05 M Ce-MBGN, and 0.2 M Ce-MBGN, and (c) TEM images of MBGN, 0.05 M Ce-MBGN, and 0.2 M Ce-MBGN. MBGN, mesoporous bioactive glass nanoparticle; SEM, scanning electron microscope; TEM, transmission electron microscopy.

To incorporate Ce into MBGNs, an adapted postimpregnation method previously reported was performed [41,42]. Briefly, the as-synthesized MBGNs were soaked in an ethanol solution of cerium nitrate (0.2M or 0.05M) at the concentration of 10 ​mg/mL under stirring for 24 ​h at different temperatures (i.e., 20, 60, and 80 ​°C). After the impregnation process, the treated MBGNs were washed with ethanol twice before drying at 60 °C overnight. The dried nanoparticles were then calcined in air at 680 ​°C for 2 ​h with a heating rate of 2 ​°C/min in a furnace. The unmodified MBGNs were also calcined at 680 ​°C for comparison. Fig. 1a shows the schematic illustration of the MBGN synthesis and the postmodification process.

### 2.4 Physicochemical characterization of MBGNs and Ce-MBGNs

The morphology of particles and their surface microstructure were characterized using field emission scanning electron microscope (SEM; Auriga, Zeiss, Germany) and transmission electron microscopy (TEM; Phillips CM30). For the SEM observation, the samples were dispersed in ethanol by ultrasonication and then dropped on conductive aluminum tapes without sputter coating. SEM images were taken at accelerating voltages between 1.5 ​kV and 3 ​kV. For TEM observation, the particles were ultrasonically dispersed in ethanol and dropped on Cu grids. TEM images were then taken at an accelerating voltage of 300 ​kV.

The composition of nanoparticles was analyzed using energy dispersive spectroscopy (EDS, X-Max^N^ Oxford Instruments, UK) at an accelerating voltage of 15 ​kV and a working distance of 6 ​mm during SEM imaging. In addition, inductively coupled plasma atomic emission spectroscopy (ICP-AES, SPECTRO CIROS-CCP spectrometer) was also used to determine the chemical composition of the particles. For ICP-AES measurement, the samples were digested by using microwave heating (heating from room temperature to 180 °C in 5 ​min and held for 5 ​min, then a second ramp up to 230 °C in 5 ​min and held for 20 ​min, followed by cooling down to room temperature), and 10 ​mL of concentrated HF-HNO_3_-HCl mixture in 1:1:3 ​vol ratio was used as the digestion medium. The resulting samples were diluted to 100 ​mL with deionized water for the analysis.

The zeta-potential of samples was measured using a Zetasizer Nano ZS (Malvern Instruments, UK) instrument with a 4 ​mW HeNe laser (633 ​nm) and a light scattering detector positioned at 90°. Hydrodynamic particle size and polydispersity index (PDI) were examined under dynamic light scattering (DLS, Zetasizer Nano ZS) at 25 ​°C, setting a minimum of 10 and a maximum of 100 runs per measurement. For the measurements, the samples were dispersed in DPBS at a concentration of 1 ​mg/mL. The analyses were performed in triplicate.

Fourier-transform infrared spectroscopy (FTIR) analysis of all samples was carried out in attenuated total reflectance (ATR) mode by using the IRAffinity-1S (Shimadzu, Japan) spectrophotometer with a resolution of 4 ​cm^−1^ and 40 scans in the wavenumber range of 2000–400 ​cm^−1^. The spectra were normalized to maximum absorption at 1056 ​cm^−1^. Powder X-ray diffraction (XRD) was performed using a Philips X'pert diffractometer (Philips, Netherlands) in the 2Ɵ range of 20–80° with Cu *Kα* radiation. A step size of 0.020° with a dwelling time of 1 ​s per step was applied.

UV–Vis (ultravioet-visible) absorption spectra of the samples were obtained by means of a Cary 5000 UV–vis-NIR spectrophotometer (Agilent, USA) to determine the absorption spectra in the range of 350–800 ​nm with BaSO_4_ as reference under ambient conditions. High-resolution X-ray photoelectron spectroscopy ​spectra of samples were recorded with a Thermo Scientific ESCALAB 250Xi spectrometer using monochromatic Al Kα X-rays under a vacuum of 5 ​× ​10^−10^ ​Torr or less. The energy resolution was set to 1 ​eV/step at ​pass energy of 187.85 ​eV for survey scans and 0.1 ​eV/step and 29.35 ​eV pass energy for high-resolution region scans. Data analysis was performed by using CasaXP software (Casa Software Ltd, UK).

SSA ​and pore structure of the samples were determined by using nitrogen adsorption-desorption analysis on ASAP2020 (Micromeritics, USA). The samples were outgassed at 150 °C for 3 ​h before the measurement. The SSA of samples was calculated using the Brunauer-Emmett-Teller ​method in the range of relative pressure 0.04–0.2 while the pore size distribution was evaluated through the density functional theory ​(DFT) method using the non-local density functional theory (NLDFT) equilibrium model for cylindrical pores.

### 2.5 Ion release behavior

To evaluate the ion release behavior of MBGNs and Ce-MBGNs, 20 ​mg of particles was placed in 20 ​mL of DPBS (pH ∼7.4 ​at 25 ​°C) in an incubator (KS 4000i control, IKA, Germany) for up to 14 ​d at 37 °C and 120 ​rpm. At each predetermined time point, 10 ​mL of the supernatant was collected by centrifugation and filtration, and the samples were replenished with 10 ​mL of fresh DPBS. The collected supernatants were then analyzed using ICP-AES to determine the concentration of released Si, Ca, and Ce ions.

### 2.6 Cell culture

The murine fibroblast cell line L929, murine macrophages J774a.1, and osteoblast-like SAOS-2 ​cells (European Collection of Cell Cultures) were used in this study. Fibroblast cells were cultured in MEM supplemented with 10% FBS, streptomycin (100 ​g/L), penicillin (100 U/mL), and 2 ​mmol/L l-glutamine at 37 °C in a humidified incubator equilibrated with 5% CO_2_. Cells were harvested before confluence by using a sterile trypsin-EDTA solution (0.5 ​g/L trypsin, 0.2 ​g/L EDTA in phosphate-buffered saline (PBS), pH 7.4) and resuspended in the experimental cell culture medium to 1 ​× ​10^5^ ​cells/mL. The macrophages were cultured in DMEM supplemented with 10% FBS, penicillin (100 U/mL), streptomycin (100 ​μg/mL), and 4 ​mM l-glutamine, at 37 ​°C in a 100% humidified incubator equilibrated with 10% CO_2_. The cells were passaged 2–3 days before use. SAOS-2 ​cells were cultured in MEM supplemented with 10% FBS, streptomycin (100 ​g/L), penicillin (100 U/mL), and 2 ​mmol/L l-glutamine, at 37 °C in a humidified incubator equilibrated with 5% CO_2_. The cell suspension was obtained by adding 2 ​mL of a sterile 0.5% trypsin-EDTA solution and resuspended in the experimental culture medium to 1.45 ​× ​10^5^ ​cells/mL. Two experimental approaches were used for cell tests in this study, that is, a direct contact method in which cells were cultured directly with the samples, and an indirect method in which the particle suspensions were placed in a Transwell® membrane insert (<0.3 ​μm, Sarstedt, Germany) during the cell culture.

### 2.7 *In vitro* cytotoxicity

In the preliminary qualitative test, fibroblast cells were cultured with the sterile samples (sterilized by heating at 160 °C for 3 ​h) at a concentration of 1 ​mg/mL for 72 ​h. The cell morphology was then observed by inverted microscope. In the quantitative test, the viability of cells cultured with the sample suspension in a Transwell® membrane insert was assessed by using the MTT assay. Briefly, the fibroblast cells were seeded on 12-well polystyrene plates (Falcon™, Thermo Fisher Scientific, Waltham, USA) underneath the Transwell® insert containing 1 ​mg/mL of particle suspension. After 72 ​h of culture, 1 ​mL of the MTT solution (5 ​mg/mL) was added to the culture medium. After ​further incubation for 3 ​h at 37 °C, the formed formazan was then dissolved in 1 ​mL dimethylsulphoxide and the absorbance was spectrophotometrically measured at 562 ​nm on a microplate spectrophotometer. The measured optical density was used to calculate cell viability. The cells cultured on the polystyrene plate without the addition of samples were used as the negative control, whereas the cells cultured in the presence of 20 ​μL of NPS solution (0.08 ​mg/mL) were identified as the positive apoptotic control.

### 2.8 Expression of proinflammatory genes

The inflammatory response of MBGNs and Ce-MBGNs was investigated with a direct contact method. Macrophages were seeded in 24-well polystyrene plates containing the nanoparticles (1 ​mg/mL) at a density of 2 ​× ​10^4^/mL. After 4 ​h of culture, the RNA from cells was isolated by using the Maxwell® RSC simply RNA Cells Kit (Promega, Thermo Fisher Scientific, Waltham, USA) and reverse transcribed by the High-Capacity cDNA Reverse Transcription Kit (Applied Biosystems, Thermo Fisher Scientific, Waltham, USA). The real-time reverse transcription polymerase chain reaction (RT-qPCR) was performed on the Applied Biosystems QuantStudio 5 real-time polymerase chain reaction (PCR) system (Thermo Fisher Scientific, Waltham, USA). Mouse interleukin-1β (IL-1β), interleukin-6 (IL-6), tumor necrosis factor alpha (TNFα), and tyrosine 3-monooxygenase/tryptophan 5-monooxygenase activation protein zeta (YWHAZ) were chosen from the collection of the TaqMan® Gene Expression Assays.

The PCR primers used were obtained from Applied Biosystems, and TaqMan® probes assay IDs are listed in Table 1. RT-qPCR was performed in duplicate for all samples according to the manufacturer's instructions. The content of cDNA samples was normalized through the comparative threshold cycle (ΔΔCt) method, consisting of the normalization of the number of target gene copies versus the endogenous reference gene YWHAZ.

**Table 1:** RT-qPCR TaqMan® probes assay IDs applied in this study.

Genes	Primers obtained from Applied Biosystems
IL-1β	Mm01336189_m1
IL-6	Mm99999064_m1
TNFα	Mm00443258_m1
YWHAZ	Mm03950126_s1
GAPDH	Hs00266705_g1
COL1A1	Hs00164004_m1
RANKL	Hs00243519_m1
SPARC	Hs00234160_m1
OPG	Hs00900358_m1
ALPL	Hs01029144_m1
NOS2	Mm00440502_m1
PTGS2	Mm00478374_m1
NRROS	Mm00524817_m1
MMP9	Mm00442991_m1

ALPL, alkaline phosphatase; IL-1β, interleukin-1β; IL-6, interleukin-6; TNFα, tumor necrosis factor alpha; RT-qPCR, reverse transcription polymerase chain reaction; YWHAZ, tyrosine 3-monooxygenase/tryptophan 5-monooxygenase activation protein zeta; GAPDH, glyceraldehyde-3-phosphate dehydrogenase, COL1A1, collagen 1A1; RANKL, receptor activator of nuclear factor kappa-Β ligand; SPARC, secreted protein acidic and cysteine rich; OPG, osteoprotegerin; NOS2, nitric oxide synthase 2; PTGS2, cyclooxygenase 2; NRROS, negative regulator of reactive oxygen species; MMP9, matrix metalloproteinase 9.

### 2.9 Expression of oxidative stress–related genes

The oxidative stress response of macrophages induced by MBGNs and Ce-MBGNs was evaluated using the direct contact method. In brief, macrophages (2.2 ​× ​10^5^/mL) were seeded onto 12-well tissue culture polystyrene plates containing the particles at the concentration of 1 ​mg/mL. After 12 ​h of incubation, 20 ​μL of NPS solution (40 ​mg/mL) was added to the medium to reach a final concentration of 400 ​μg/mL per well. After further 4-h or 48-h incubation of culture, the RNA from macrophages was isolated by using the Maxwell® RSC simply RNA Cells Kit and reverse transcribed by the High-Capacity cDNA Reverse Transcription Kit. Mouse nitric oxide synthase 2 (NOS2), cyclooxygenase 2 (PTGS2), negative regulator of reactive oxygen species (NRROS), matrix metalloproteinase 9 (MMP9), and YWHAZ were selected from the collection of the TaqMan Gene Expression Assays. The primers used are listed in Table 1. RT-qPCR was performed in duplicate according to the manufacturer's instructions. The content of cDNA samples was normalized using the ΔΔCt method, consisting of the normalization of the number of target gene copies versus the endogenous reference gene YWHAZ.

### 2.10 Expression of osteogenesis-related genes

Osteoblast-like SAOS-2 ​cells were cultured in 12-well tissue polystyrene plates at a density of 1.45 ​× ​10^5^ ​cells/mL while each well contained the Transwell® insert with nanoparticles added at the concentration of 1 ​mg/mL. After either 72 ​h or 7 ​d of incubation, the expression of glyceraldehyde-3-phosphate dehydrogenase (GAPDH), collagen 1A1 (COL1A1), receptor activator of nuclear factor kappa-Β ligand (RANKL), secreted protein acidic and cysteine rich (SPARC), osteoprotegerin (OPG), and alkaline phosphatase (ALPL) genes, as cell osteogenic differentiation markers, was assessed by RT-qPCR in duplicate according to the manufacturer's instructions. The primers used are listed in Table 1. The RNA from SAOS-2 ​cells was isolated by using the Maxwell® RSC simply RNA Cells Kit. RNA was reverse transcribed by the High-Capacity cDNA Reverse Transcription Kit, and RNA quantitation was performed before starting the RT-qPCR in the Quantus Fluorometer (Promega, Thermo Fisher Scientific, Waltham, USA), by using the Quantifluor system kit (Promega, Thermo Fisher Scientific). The content of cDNA samples was normalized by using the ΔΔCt method.

### 2.11 Statistical analysis

Experimental data are reported as mean ​± ​standard deviation. Statistical differences between groups were analyzed using the two-way analysis of variance (ANOVA) statistical test with Tukey's post-hoc test and the one-way ANOVA with Tukey's pairwise post-hoc test. Statistical significance is represented as *p ​< ​0.05, **p ​< ​0.01, ***p ​< ​0.001, and ****p ​< ​0.0001.

## 3 Results

### 3.1 Optimization of Ce-MBGN synthesis using the postmodification approach

Ce-MBGNs were synthesized by using a two-step strategy, where MBGNs (nominal composition 70SiO_2_–30CaO, in mol%) were first synthesized using a microemulsion-assisted sol-gel method as reported in the literature [40], followed by a postimpregnation approach to incorporate Ce into MBGNs. The as-synthesized MBGNs appeared to be spherical showing surface nanopores (Fig. 1b). In addition, ellipse-shaped particles could also be observed, which were likely induced because of the fusion of microemulsion templating droplets during synthesis [40]. The size of MBGNs was in the range of 100–300 ​nm as seen in SEM images (Fig. 1b), which was in good agreement with the size of particles synthesized using similar microemulsion-based approaches reported in the literature [40,42]. The TEM image of MBGNs (Fig. 1c) confirms the presence of mesopores throughout the nanoparticles. The pores were not fully homogeneous in size and structure, most likely resulting from the interactions occurring between cationic Ca ions and cationic surfactant CTAB molecules during the reaction, which consequently induced a disturbing effect on the micelle self-assembly to form the mesophases [4]. EDS spectrum of MBGNs (Fig. S1) proves the presence of Si and Ca in the nanoparticles, and their chemical composition was calculated to be (86.1 ​± ​0.3)SiO_2_-(13.9 ​± ​0.2)CaO (mol%) based on the atomic ratio obtained.

In the impregnation process, we first evaluated the effect of the impregnation temperature (20, 60, and 80 ​°C) on the incorporation yield, for which a fixed concentration of cerium nitrate in ethanol (0.2M) was used. EDS results (Fig. S2) showed that the amount of incorporated Ce increased by raising the treating temperature. The molar concentrations of Ce in the particles were calculated to be ∼2, 13, and 18% for the particles modified at 20, 60, and 80 ​°C, respectively (Fig. S1 and Fig. S2). However, higher temperatures appeared to facilitate the formation of nanoceria clusters. SEM images of the particles treated at different temperatures (Fig. S2) showed the presence of nanoclusters in the nanoparticles impregnated at 60 and 80 °C at variance with those treated at 20 ​°C. XRD results (Fig. S2) of the nanoparticles modified at 60 and 80 ​°C confirmed the formed nanoclusters being crystalline nanoceria. The formation of nanoceria for the samples treated at higher temperatures was likely because the procedure increased nucleation kinetics [43]. Although nanoceria also possesses remarkable antioxidant properties [44], their absence in Ce-MBGNs is preferred considering further applications (e.g., drug delivery and nanocomposite fabrication) where chemical homogeneity and particle dispersity are of high interest. Therefore, we selected 20 ​°C as the impregnation temperature for further study.

The influence of Ce precursor solution's concentration on the incorporation yield was then evaluated. Fig. 1b shows SEM images of MBGNs modified in 0.05M and 0.2M cerium nitrate ethanol solution at 20 ​°C (referred to as 0.05 M Ce-MBGN ​and 0.2 M Ce-MBGN, respectively), in which the nanoparticles appeared to retain their shape and size while no significant particle aggregation and ceria nanoclusters were observed. TEM images (Fig. 1c) further evidence that the nanoparticles retained their morphology. In addition, the mesoporous structure was preserved after the modification, and no nanoceria particles were formed inside MBGNs as shown in TEM images. The presence of Ce in the modified particles was confirmed by EDS results (Fig. S1). According to the EDS results, the chemical compositions of 0.05 M Ce-MBGN and 0.2 M Ce-MBGN were estimated to be (86.6 ​± ​0.6)SiO_2_-(12.1 ​± ​0.4)CaO-(1.3 ​± ​0.2)CeO_2_ (mol%) and (86.0 ​± ​0.5)SiO_2_-(11.8 ​± ​0.8)CaO-(2.2 ​± ​0.3)CeO_2_ (mol%), respectively. As expected, the amount of incorporated Ce increased with increasing the concentration of Ce solution for impregnation. The chemical compositions of both Ce-MBGNs were also analyzed by using ICP-AES after powder dissolution. Results showed that the concentrations of Ce in 0.05 M Ce-MBGN and 0.2 M Ce-MBGN were (1.8 ​± ​0.2)% CeO_2_ and (2.8 ​± ​0.4)% CeO_2_ (mol%), respectively, slightly higher than those estimated from the EDS results. Overall, the results indicated that the amount of incorporated Ce could be controlled by tuning the concentration of the impregnation solution. For further studies, 0.05 M Ce-MBGN and 0.2 M Ce-MBGN obtained at 20 ​°C were selected.

### 3.2 Physicochemical characterization of Ce-MBGNs

**Fig. 2:**
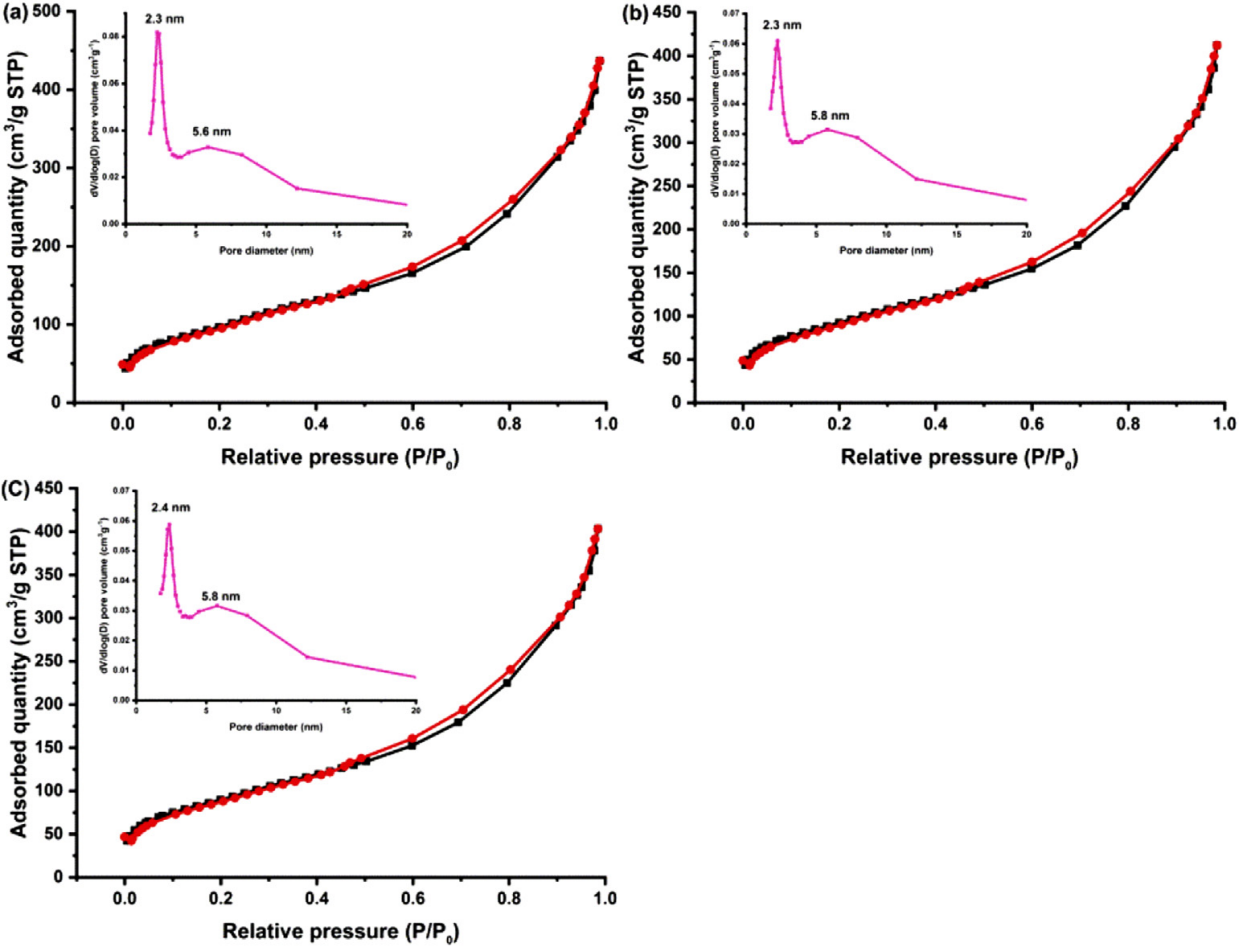
N_2_ adsorption-desorption isotherms and inserted pore size distribution curves for (a) MBGN, (b) 0.05 M Ce-MBGN, and (c) 0.2 M Ce-MBGN. MBGN, mesoporous bioactive glass nanoparticles; Ce-MBGN, cerium-containing MBGN.

N_2_ adsorption-desorption isotherms of the obtained MBGNs and Ce-MBGNs (Fig. 2) exhibit type IV isotherm characteristics as defined by International Union of Pure and Applied Chemistry (IUPAC) [45], which is a typical isotherm for mesoporous materials. Moreover, MBGNs showed a heterogeneous pore size distribution, which exhibited a narrow pore size distribution centered at 2.3 ​nm and a wide distribution centered at 5.6 ​nm (Fig. 2a). The relatively larger pores discernible in the corresponding TEM image (Fig. 1c) were probably induced by the volatilization of ethyl acetate enlarging the pores during calcination [46]. After the incorporation of Ce, the particles maintained the narrow pore size distribution centered at ∼2.3 ​nm and the wide distribution (Fig. 2b and c). However as seen in Table 2, the SSA of particles was slightly reduced from 381 ​m^2^/g (MBGNs) to 360 ​m^2^/g (0.05 M Ce-MBGN) and 344 ​m^2^/g (0.2 M Ce-MBGN), probably because of the occlusion of a fraction of nanopores during modification. The pore volume (Table 2) of particles (0.7, 0.7, and 0.6 ​cm^3^/g for MBGNs, 0.05 M Ce-MBGN, and 0.2 M Ce-MBGN, respectively) was not significantly affected by modification. Table 2 also shows the zeta potential and PDI of the nanoparticles in PBS. All particles exhibited negative ​surface charge as expected, considering that the isoelectric point ​of silica-based nanoparticles is ∼2 [39]. No significant difference in zeta potential was observed among the nanoparticles, but the PDI increased from 0**.**153 (MBGNs) to 0.228 for 0.05 M Ce-MBGN ​and 0.278 for 0.2 M Ce-MBGN ​after the incorporation of Ce, indicating a slight reduction in particle dispersity.

**Table 2:** Physicochemical characterization results for MBGNs and Ce-MBGNs.

Materials	Zeta potential (mV)	Hydrodynamic sizes (nm)	PDI	Specific surface area (m^2^/g)	Pore volume (cm^3^/g)
MBGN	−22 ​± ​1	247 ​± ​5	0.153	381	0.7
0.05 M Ce-MBGN	−21 ​± ​2	263 ​± ​6	0.228	360	0.7
0.2 M Ce-MBGN	−22 ​± ​2	256 ​± ​4	0.278	344	0.6

MBGN, mesoporous bioactive glass nanoparticle; PDI, polydispersity index; Ce-MBGN, cerium-containing MBGN.

**Fig. 3:**
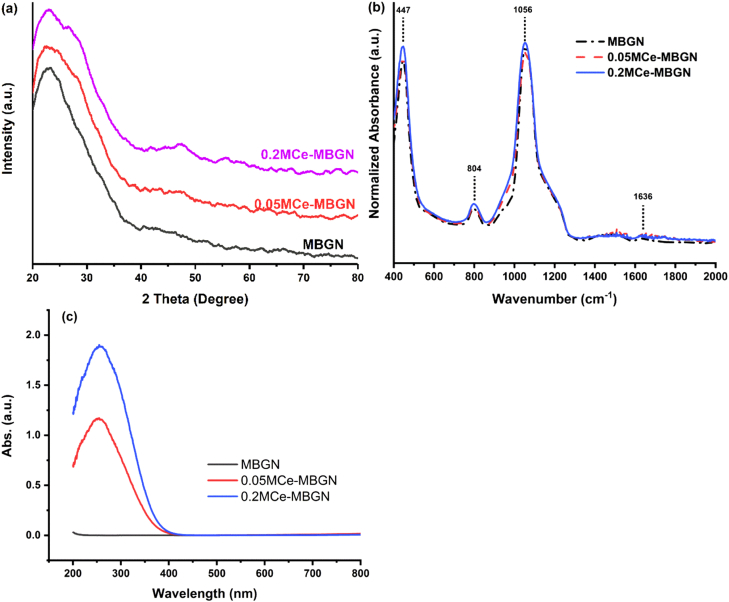
(a) XRD patterns, (b) ATR-FTIR spectra, and (c) UV–Vis spectra of MBGN and Ce-MBGN. XRD, X-ray diffraction; ATR-FTIR, attenuated total reflectance–Fourier-transform infrared spectroscopy; MBGN, mesoporous bioactive glass nanoparticles; Ce-MBGN, cerium-containing MBGN; UV-Vis, ultraviolet-visible.

XRD patterns (Fig. 3a) confirmed the amorphous structure of MBGNs, as only a broad reflection between 2Ɵ ​= ​20 and 30° could be observed, which is the typical XRD pattern of amorphous silicate materials [47]. After postmodification, no obvious diffraction peaks could be observed in the XRD patterns of Ce-MBGNs. However, a weak shoulder located at 2Ɵ ∼29° and a broad band located at 2Ɵ ∼47°, which could be assigned to the (111) and (220) crystallographic planes of nanoceria (JCPDS 34-0394) [48], could be observed in the patterns of 0.2 M Ce-MBGN. The detection of these bands suggested that 0.2 M Ce-MBGN could contain a minimal amount of nanoceria, but large domains of nanoceria were not induced. In addition, the presence of nanoceria could not be observed in SEM and TEM images (Fig. 1). Fig. 3b shows normalized ATR-FTIR spectra of the particles before and after the incorporation of Ce. Characteristic bands of silicate glasses located at 447 ​cm^−1^ (Si–O–Si rocking), 804 ​cm^−1^ (Si–O–Si bending), and 1056 ​cm^−1^ (Si–O–Si stretching) [49] could be observed in the spectra of all the nanoparticles. A band located at 1636 ​cm^−1^ related to adsorbed molecular water was also observed in all FTIR spectra [50]. After surface modification, the relative intensity of these bands slightly increased. However, no obvious new bands related to Si–O-non-bridging oxygen were observed after surface modification, perhaps because of the low concentration of incorporated Ce not significantly changing the silicate structure.

**Fig. 4:**
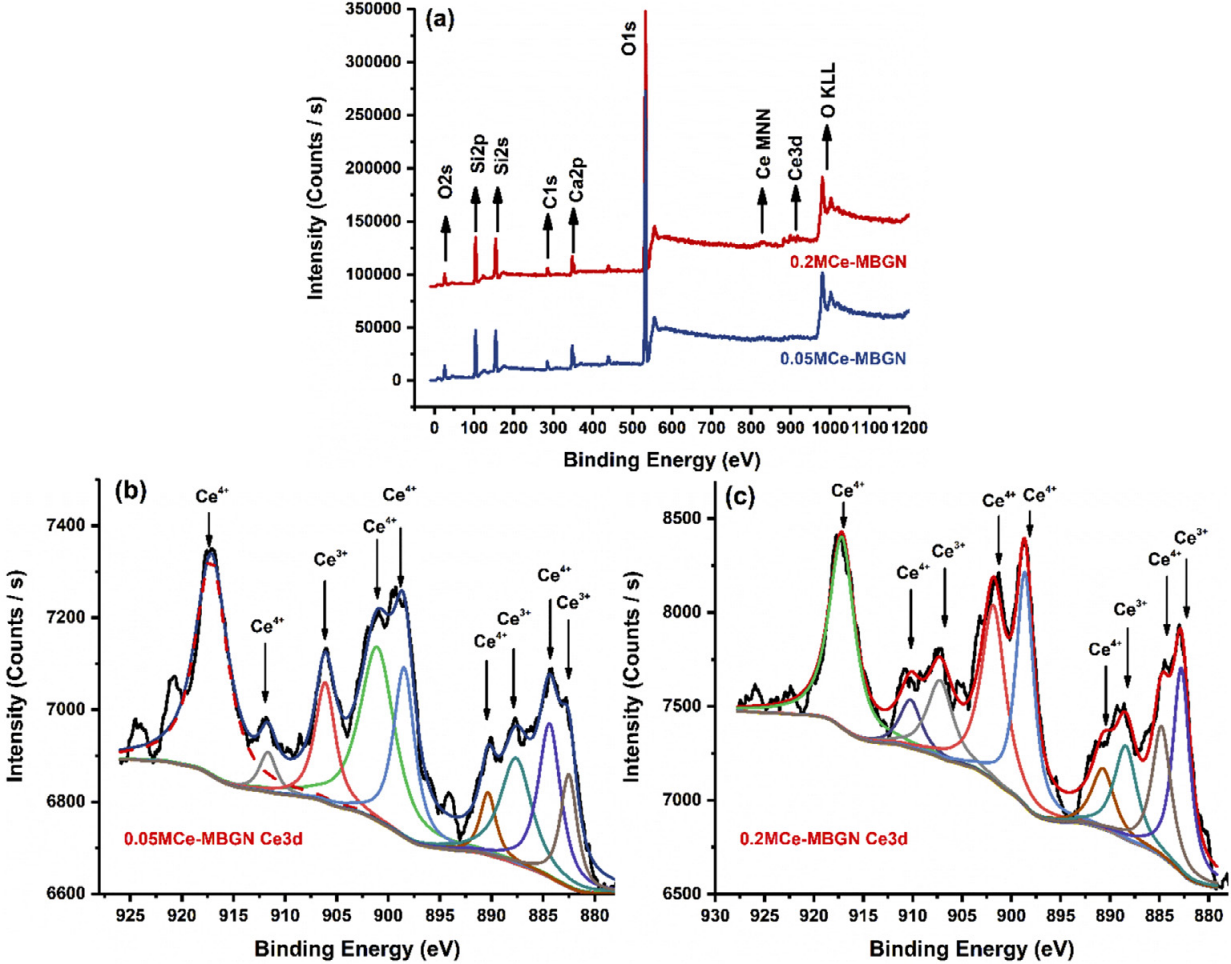
(a) XPS survey spectra for 0.05 M Ce-MBGN and 0.2 M Ce-MBGN; Ce3d deconvoluted photoelectron spectra for (b) 0.05 M Ce-MBGN and (c) 0.2 M Ce-MBGN. MBGN, mesoporous bioactive glass nanoparticles; Ce-MBGN, cerium-containing MBGN; XPS, X-ray photoelectron spectroscopy.

UV–vis adsorption spectra (Fig. 3c) of the nanoparticles also proved the presence of Ce in both types of Ce-MBGNs. MBGNs showed no specific adsorption bands in the range of 200–800 ​nm, as the tetrahedral SiO_4_ structure in MBGNs does not adsorb light in this range [51]. After modification, a broad adsorption band located between ∼200 and 350 ​nm was observed. In this range, both Ce^3+^ and Ce^4+^, due to the charge transfer transition O^2−^ - Ce^3+^ and O^2−^ - Ce^4+^, respectively [51,52], could exhibit specific adsorption bands. The observation of such broad bands in both types of Ce-MBGNs could be ascribed to the compresence of both Ce^3+^ and Ce^4+^ species, which results in overlapped adsorption bands [23]. Moreover, the intensity of this band was stronger in 0.2 M Ce-MBGN than in 0.05 M Ce-MBGN, suggesting a higher concentration of Ce in 0.2 M Ce-MBGN. XPS results (Fig. 4) were in line with the EDS and UV–vis results. The survey scan of XPS (Fig. 4a) confirmed the presence of Si, Ca, and Ce on the surface of the particles. The high resolution XPS spectra of Ce3d (Fig. 4b and c) were deconvoluted following the Neal Fairley guidelines [36]. After the deconvolution, both Ce^3+^ and Ce^4+^ oxidation states could be highlighted in the spectra [36,53], which was consistent with the UV–Vis results. In addition, the intensity of these Ce-related peaks was higher in 0.2 M Ce-MBGN, indicating the higher concentration of Ce in these particles. Notably, although 0.05 M Ce-MBGN and 0.2 M Ce-MBGN contained different amounts of Ce, the relative molar percentage of Ce^4+^ prevailed in both particles and was similar (∼74%).

### 3.3 Ion release behavior of Ce-MBGNs

**Fig. 5:**
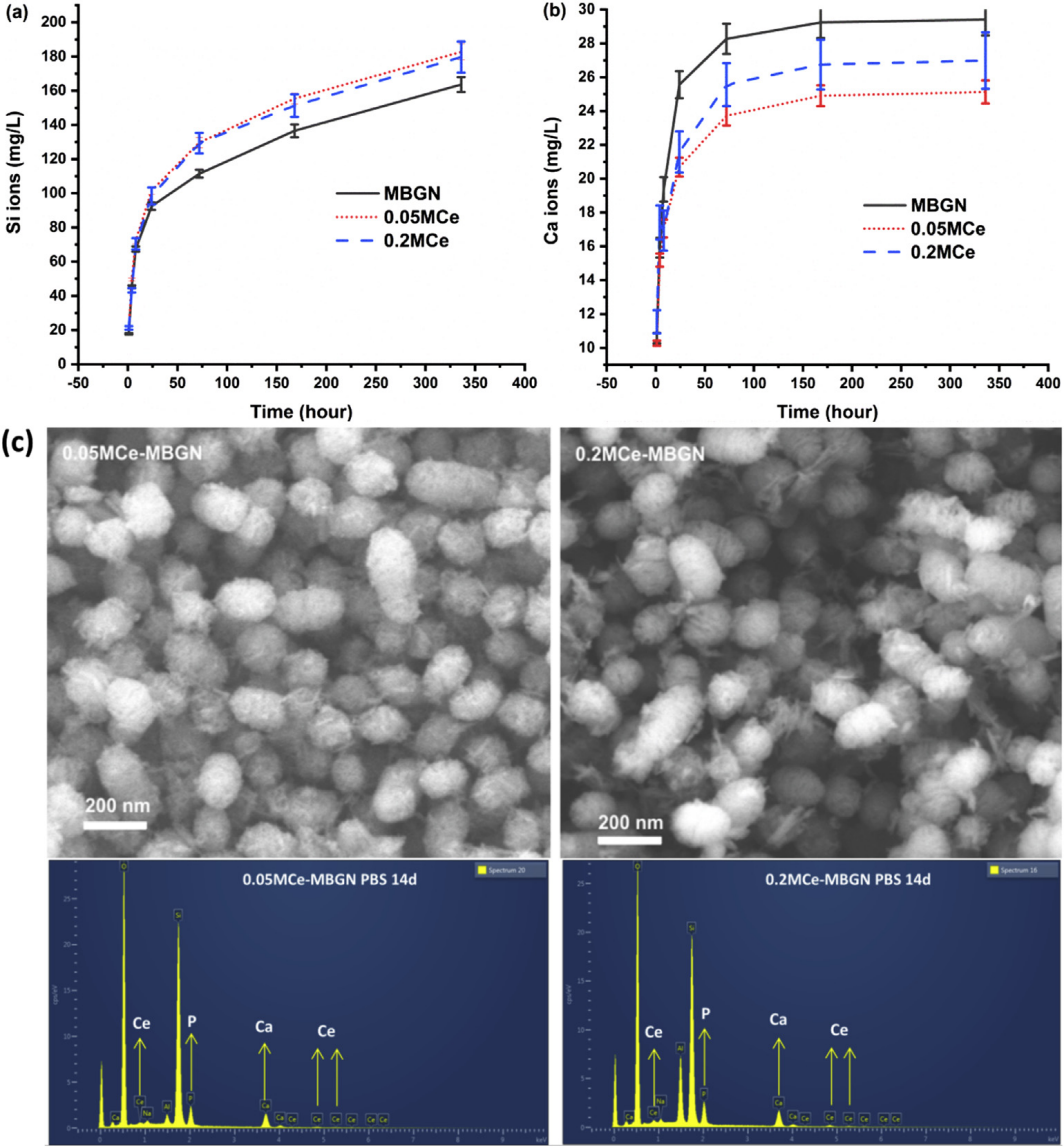
(a-b) Ion release profiles of MBGN, 0.05 M Ce-MBGN, and 0.2 M Ce-MBGN in DPBS; (c) SEM images of EDS spectra of 0.05 M Ce-MBGN and 0.2 M Ce-MBGN after immersion in DPBS for 14 ​d. MBGN, mesoporous bioactive glass nanoparticles; Ce-MBGN, cerium-containing MBGN; EDS, energy dispersive spectroscopy; SEM, scanning electron microscope; DPBS, Dulbecco's phosphate-buffered saline.

Understanding the dissolution behavior of Ce-MBGNs is essential for their further biomedical applications. Fig. 5 shows the ion release profiles of MBGNs, 0.05 M Ce-MBGN, and 0.2 M Ce-MBGN in DPBS for up to 14 ​d. All particles were seen to exhibit a stable and sustained release of Si ions, suggesting the dissolution of these silica-based nanoparticles under physiological conditions. Both Ce-MBGNs appeared to release a slightly larger amount of Si ions than MBGNs while no significant difference could be observed between themselves. Such a phenomenon could be induced because of the slightly higher solubility of Ce-MBGN as a result of the incorporation of Ce. In addition, the impregnation process could partially break the silicate structure of the nanoparticles [51]. Unlike the release of Si ions, all nanoparticles showed a burst release of Ca ions within 24 ​h followed by a stable ion release (Fig. 5b). MBGNs released, as expected, a larger amount of Ca ions than Ce-MBGNs because of the higher concentration of CaO in their framework. However, further release of Ca ions seemed to be retarded after 3 ​d for all the nanoparticles, which could be ascribed to their high surface reactivity in biological fluids (e.g., SBF, PBS) with the precipitation of calcium phosphate layers that hinder future ion exchange reactions [47]. Notably, no significant release of Ce ions was detected, which has also been observed in other sol-gel–derived Ce-BGs [33,35]. It is known that Ce ions can form insoluble complexes in the presence of phosphate groups [35,47]. Thus, the released Ce ions from Ce-MBGNs might interact with phosphate groups to form precipitates at the nanoparticle surface instead of diffusing into the soaking solution [47]. The ion release behavior of Ce-MBGNs might vary in different physiological fluids, but the interaction between Ce and phosphate groups would generally occur, given the abundance of these groups in body fluids. Fig. 5c shows SEM images and EDS spectra of both Ce-MBGNs after immersion in DPBS for 14 ​d. The particles still maintained the morphology after soaking, and needle-like calcium phosphate formations could also be observed. EDS spectra showed the presence of P and Ce throughout the nanoparticles, supporting the previous hypothesis related to the formation of cerium phosphate compounds and in accordance with ICP-AES measurements in the release medium [54].

### 3.4 *In vitro* cytotoxicity

**Fig. 6:**
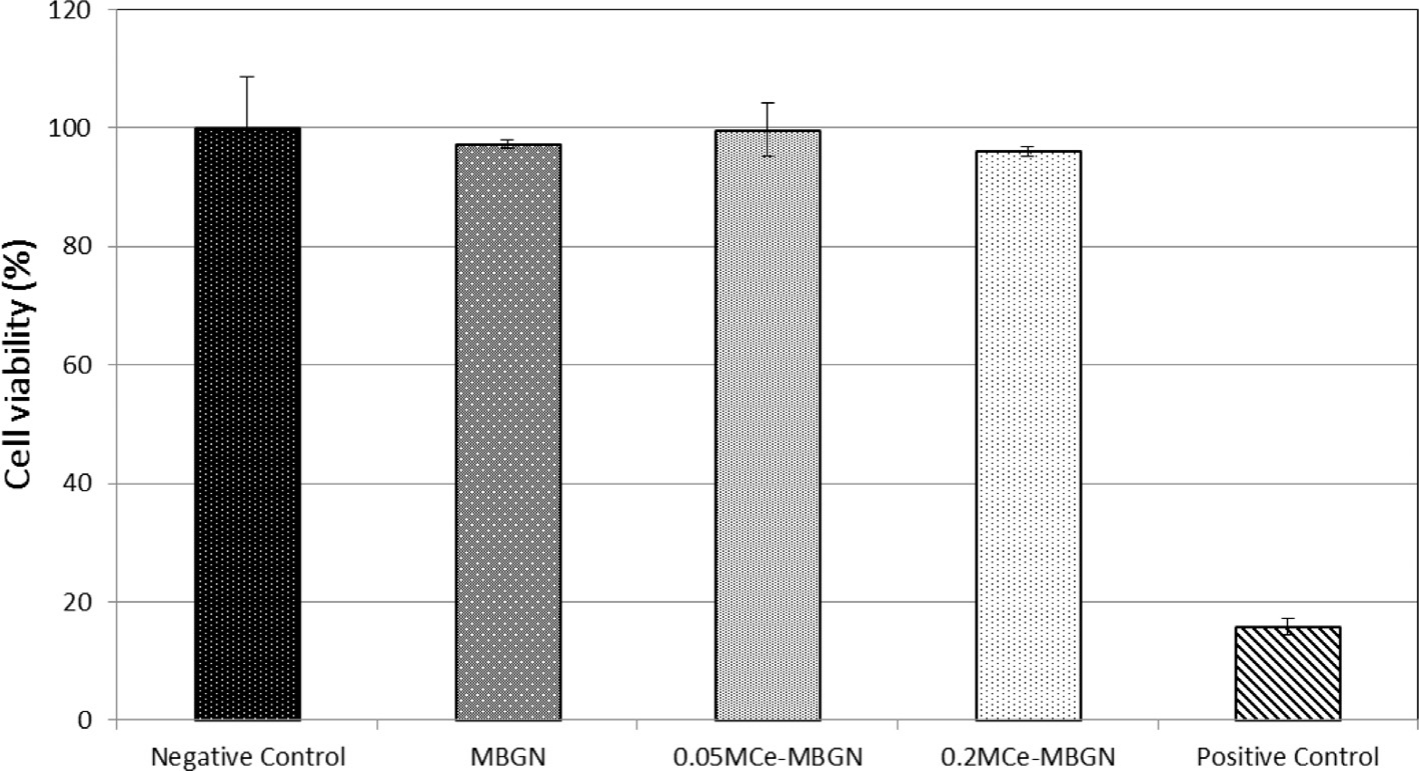
Results of MTT assay on fibroblast cells cultured with MBGN and Ce-MBGN at the concentration of 1 ​mg/mL. Fibroblast cells were selected as a model cell line to explore the biocompatibility of 0.05 M Ce- and 0.2 M Ce-MBGN by using a polystyrene plate as negative control and a polystyrene plate added with 0.08 ​mg/mL of NPS to induce cell death as the positive control. MBGN, mesoporous bioactive glass nanoparticles; Ce-MBGN, cerium-containing MBGN; MTT, 3-(4,5-dimethylthiazol-2-yl)-2,5-diphenyltetrazolium bromide; NPS, sodium nitroprusside

Fig. S3 shows representative optical images of fibroblasts cultured with MBGNs and Ce-MBGNs for 72 ​h at the concentration of 1 ​mg/mL. In comparison with the positive control group where cells were aggregated, the morphology of cells in the presence of MBGNs and Ce-MBGNs was similar to the morphology of cells grown on polystyrene plates (control), which indicated that the presence of MBGNs and Ce-MBGNs did not significantly alter the cell morphology. An indirect method was then applied to evaluate the cytotoxicity of particles. Fig. 6 shows the MTT assay results of the samples, where no significant difference among the MBGN group, Ce-MBGN group, and negative control could be observed. The obtained cell viability percentages for all the nanoparticles were far higher than 70% (in relation to the polystyrene control), that is, the required minimum value for considering the biocompatibility of the tested material, according to the international standard ISO 10993-5: 2009-Biological evaluation of medical devices: Tests for in vitro cytotoxicity. Taken together, the results indicated the non-cytotoxicity of MBGNs and Ce-MBGNs.

### 3.5 Expression of proinflammatory genes in macrophages

**Fig. 7:**
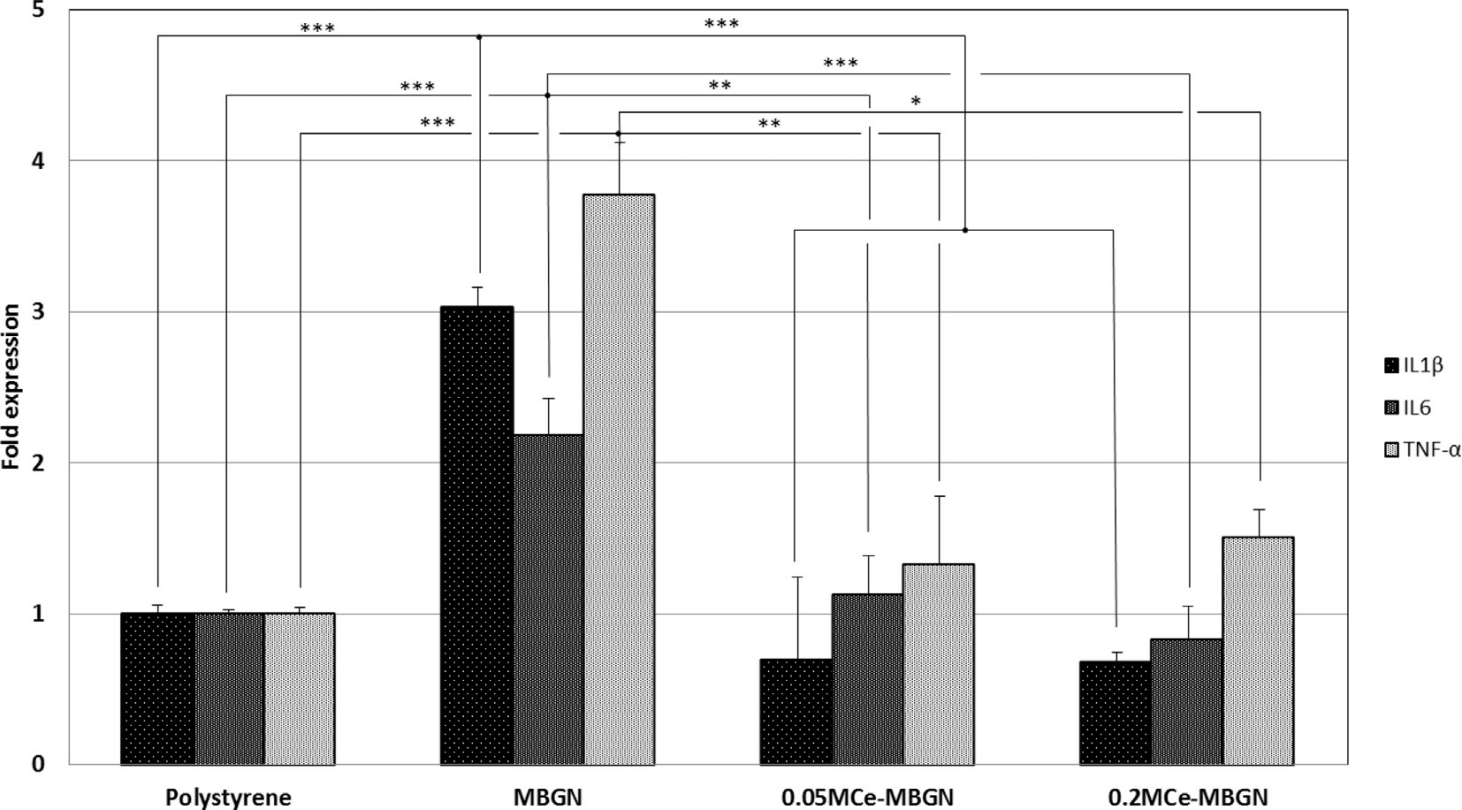
Proinflammatory gene ​expression of macrophages (J774a.1) in culture with MBGN and Ce-MBGN. MBGN, mesoporous bioactive glass nanoparticles; Ce-MBGN, cerium-containing MBGN.

Fig. 7 shows the expression of proinflammatory genes IL-1β, IL-6, and TNF-α in macrophages after culture with the nanoparticles. Compared with the control (polystyrene), MBGNs significantly upregulated the expression of all these proinflammatory genes. On the other hand, both 0.05 M Ce-MBGN and 0.2 M Ce-MBGN induced downregulation of IL-1β expression, whereas only 0.2 M Ce-MBGN downregulated the expression of IL-6. Conversely, the expression of TNF-α in both Ce-MBGN groups was slightly higher than that in the control. Notably, the expression of all the proinflammatory genes was greatly reduced in the presence of Ce-containing nanoparticles, suggesting the potential of the strategy of incorporating Ce into MBGNs to induce an anti-inflammatory response.

### 3.7 Expression of oxidative stress–related genes in macrophages

**Fig. 8:**
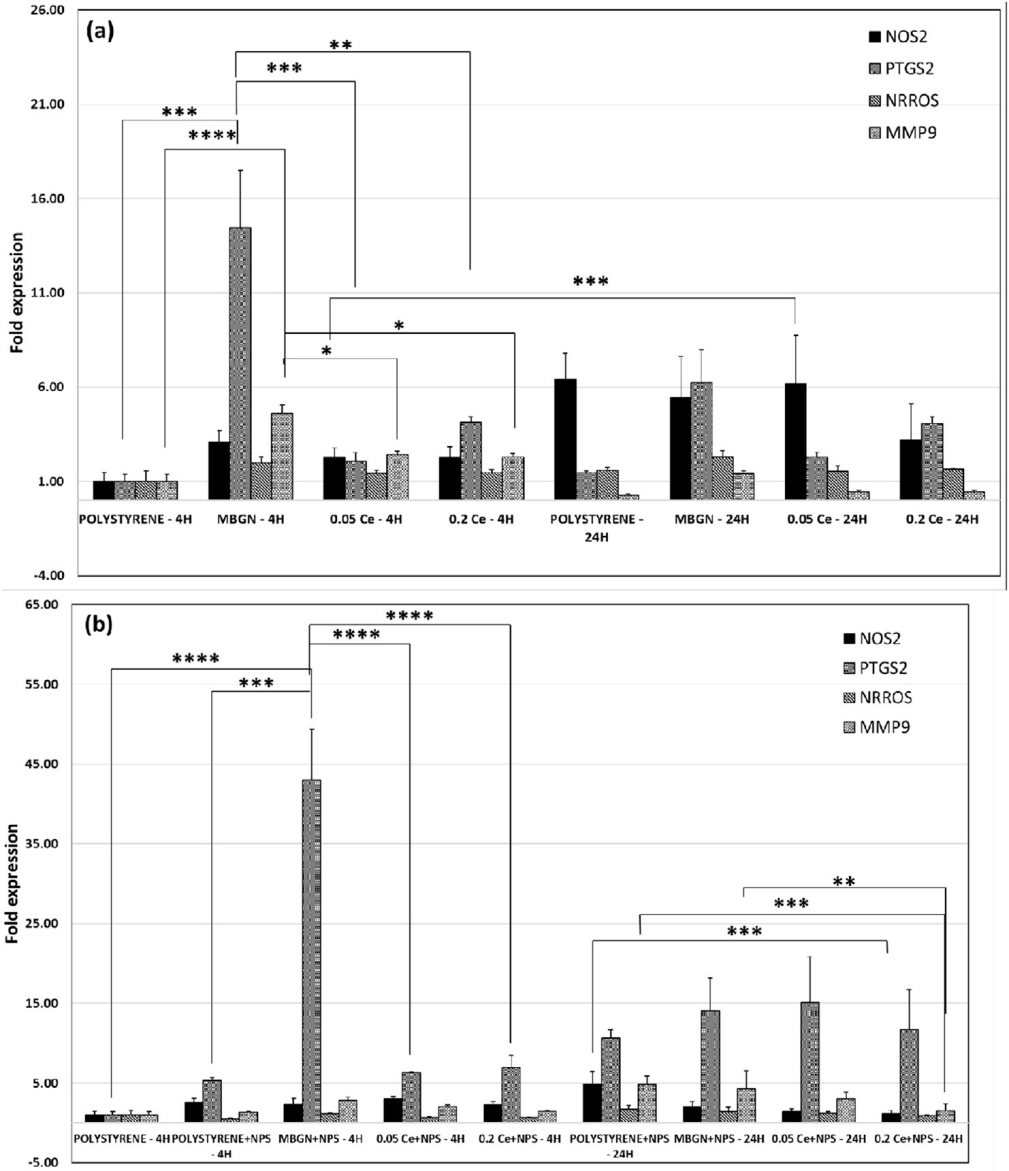
Oxidative stress–related gene expression of macrophages (J774a.1) in culture with MBGN and Ce-MBGN (a) without and (b) with the addition of oxidizing agent NPS. MBGN, mesoporous bioactive glass nanoparticles; Ce-MBGN, cerium-containing MBGN; NPS, sodium nitroprusside.

Fig. 8a shows the expression of oxidative stress–related genes NOS2, PTGS2, NRROS, and MMP9 in macrophages after culturing with the nanoparticles. In comparison with the control, an evident upregulated expression of all these genes was induced by all the nanoparticles after 4 ​h of culture, while only the expression of PTGS2 in the MBGN and 0.2 M Ce-MBGN groups and NRROS in the MBGN group was significantly upregulated after 24 ​h of culture. Notably, in comparison with the control, the 0.2 M Ce-MBGN group significantly downregulated the expression of NOS2 after 24 ​h of culture. Both types of Ce-MBGNs induced a similar expression of NRROS and MMP9 to the control after 24 ​h of culture. However, they downregulated the expression of PTGS2 and MMP9 at both time points in comparison with the MBGN group. In particular, 0.05 M Ce-MBGN appeared to downregulate the expression of PTGS2 to a greater extent than 0.2 M Ce-MBGN, but a more significant downregulation in NOS2 was induced by 0.2 M Ce-MBGN after 24 ​h of culture. We also evaluated the expression of oxidative stress–related genes in the presence of pro-oxidation agent NPS (Fig. 8b). Specifically, a significantly high expression of PTGS2 gene reaching a 43-fold change was induced by MBGNs after 4 ​h of culture in comparison with the control, while only a slight upregulation was induced by both Ce-MBGNs. On the other hand, after 24 ​h of culture, in both Ce-MBGN groups, significantly downregulated the expression of NOS2 and MMP9 was significantly downregulated in comparison with the expression in the MBGN group and the control. Interestingly, both Ce-MBGNs significantly downregulated the MMP9 and NRROS expression after 24 ​h of culture in the presence of NPS, which was, however, not observed in the case of NPS absence. The results suggested that the incorporation of Ce in MBGNs could reduce the oxidative stress responses induced by the nanoparticles. More importantly, Ce-MBGNs appeared to be able to counteract oxidant effects induced by pro-oxidant agents, such as NPS.

### 3.8 Expression of osteogenesis-related genes in osteoblast-like SAOS-2 ​cells

**Fig. 9:**
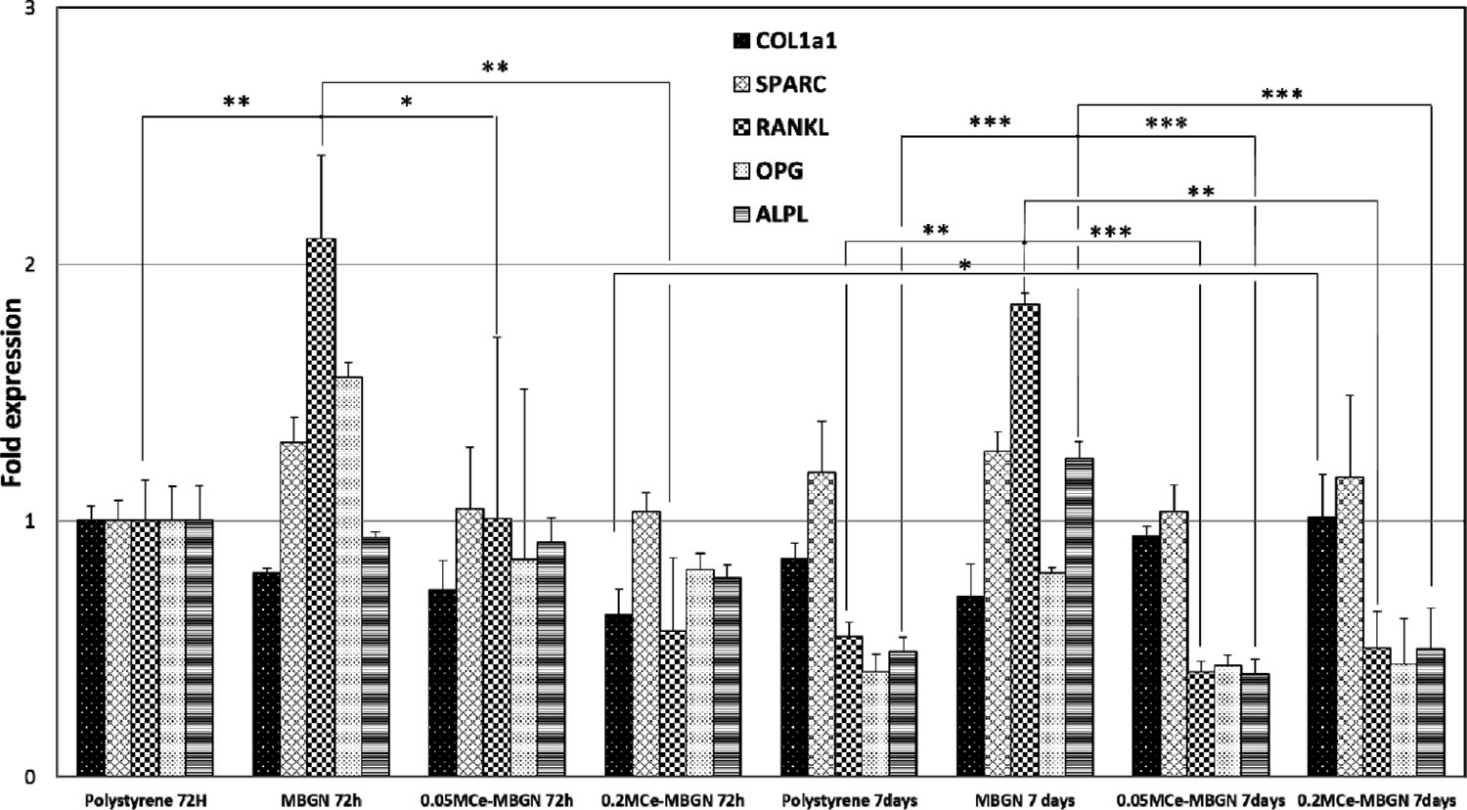
Osteogenic gene ​expression of osteoblast-like SAOS-2 ​cells cultured with MBGN and Ce-MBGN. MBGN, mesoporous bioactive glass nanoparticles; Ce-MBGN, cerium-containing MBGN.

To evaluate the effects of MBGNs and Ce-MBGNs on osteogenic activities, the expression of a set of genes was analyzed in osteoblast-like SAOS-2 ​cells after culture with the particles for 3 ​d and 7 ​d, including biomineralization-associated genes (COL1A1, ALPL), the gene related to the maturation phase of bone (SPARC), and the genes related to the bone formation/resorption equilibrium (RANKL/OPG). As shown in Fig. 9, a significantly upregulated expression of SPARC, RANKL, and OPG genes was induced by MBGNs after 3 ​d of culture, while only a slight overexpression of SPARC gene was induced by both Ce-MBGNs. However, all the particles induced a downregulated expression of COL1A1 and ALPL genes after 3 ​d of culture in comparison with the control. Furthermore, in comparison with the control, both Ce-MBGN groups inhibited the expression of OPG gene, while the MBGN group upregulated its expression. Notably, the presence of Ce in the particles induced a significant downregulation in the expression of SPARC, RANKL, and OPG genes compared with MBGNs after 3 ​d of culture, but only 0.2 M Ce-MBGN could significantly downregulate the expression of RANKL gene compared with the control. After 7 ​d of culture, a slight upregulation in the expression of COL1A1 gene was induced by both Ce-MBGN groups compared with the control and MBGN group. In addition, significant overexpression of ALPL was induced by the MBGN group after 7 ​d of culture in comparison with the control, which was not induced by both Ce-MBGN groups. The results thus confirmed that the presence of Ce in the particles could effectively induce a downregulated expression of RANKL gene, a key factor for osteoclast differentiation and activation, given the significantly upregulated expression of this gene by MBGNs.

## 4 Discussion

MBGNs are versatile building blocks for developing 3D bone scaffolds, orthopedic coatings, and composite hydrogels as well as drug delivery systems [4,55]. Ce-containing biomaterials (e.g., nanoparticles, coatings, and scaffolds) have displayed remarkable effects in relation to antioxidation, anti-inflammation, pro-osteogenesis, and proangiogenesis activities [18,[26], [27], [28]]. Therefore, the combination of BGs and Ce is an attractive strategy to develop multifunctional biomaterials for bone repair/regeneration applications [22,30,36,56]. However, the synthesis of Ce-containing BGNs still faces challenges, such as particle aggregation and formation of undesired nanoceria clusters [30,37], which negatively affect the chemical homogeneity and particle dispersity required when these nanoparticles are applied as building blocks for 3D scaffolds, coatings, or drug delivery carriers [6]. In this work, by using a postmodification method, we successfully developed chemically homogenous and highly dispersed Ce-MBGNs without the formation of nanoceria. The obtained Ce-MBGNs exhibited beneficial biological properties, such as antioxidant, anti-inflammatory, and pro-osteogenesis effects, evidencing their great potential for bone-related applications, particularly in the repair/regeneration of bone defects under inflammatory conditions.

In conventional sol-gel–based strategies for synthesizing BGNs, the incorporation of metallic ions should be carefully controlled to avoid particle aggregation and/or formation of additional side products (e.g., metallic or metal oxide nanoparticles) [4]. For example, Goh et al. [37] used a ‘quick alkali mediated’ sol-gel approach to prepare Ce-containing BGNs. However, undesired agglomeration of these particles was observed, and the presence of nanoceria was also detected when the incorporated Ce content was higher than 1 ​mol%. To overcome these drawbacks, here the incorporation of Ce into MBGNs was achieved through a postimpregnation strategy that has been widely used to introduce Ce into silica-based particles [51] and to functionalize nanoscale BGs [41,42,57]. Ethanol was selected as the impregnation solvent to minimize the dissolution of the MBGN framework during the modification process [57]. By carefully controlling the processing parameters (temperature and concentration of the soaking solution), Ce was successfully incorporated into MBGNs without significantly affecting the dispersity and mesoporous structure of the particles. Moreover, no significant formation of crystalline nanoceria was observed as evidenced by the XRD results (Fig. 3) and TEM images (Fig. 1). Although nanoceria have shown a number of beneficial ​biological activities, their presence may compromise the structural and surface properties of MBGNs, such as dissolution behavior, dispersity, and apatite-forming ability [22,39].

It is well known that the cytotoxicity of glasses is highly dependent on their chemical composition [3]. In the literature, the influence of Ce incorporated into BGs on cytotoxicity has been shown to be related to the concentration. For example, the incorporation of Ce up to 5 ​mol% into mesoporous bioactive glasses (SiO_2_–CaO–P_2_O_5_) has shown non-cytotoxicity toward ​fibroblast cells, while higher concentrations could reduce cell viability [36]. In the present study, Ce-MBGNs did not exert cytotoxic behavior against fibroblast cells. Given the Ce concentration (<5 ​mol%) in Ce-MBGNs, the results are consistent with those reported in the literature [36]. However, BGs may cause inflammatory responses even without affecting cell viability [58], which depends on the BG composition and the dosage used, as well as the type of cells tested. For instance, 45S5 Bioglass could reduce the generated IL-6 and TNF-α cytokines from activated human macrophages at relatively low concentrations [59], whereas it could increase them at relatively high concentrations [58]. Interactions between silicate BGNs and macrophages in terms of inflammatory responses have been reported [60,61], and silicate glass nanoparticles have shown their potential in modulating inflammatory responses to enhance osteogenesis and angiogenesis [61,62]. Our results (Fig. 7) evidenced that MBGNs (SiO_2_–CaO composition) could upregulate the expression of proinflammatory genes IL-1β, IL-6, and TNF-α, which is in agreement with the results previously reported [58]. Many efforts have been dedicated to minimizing the inflammatory responses induced by BGs. For this purpose, anti-inflammatory compounds (e.g., dexamethasone, polyphenols), either loaded as drugs [63] or as surface coating [21], have been combined with BGs. Inclusion of metallic ions in BGNs, such as Sr, was also confirmed to reduce the inflammatory response and to enhance osteogenesis [60,64]. Similarly, the incorporation of Ce in MBGNs was shown in this study to inhibit inflammatory responses (Fig. 7). Particularly, the expression of IL-1β and IL-6 was significantly downregulated when Ce was incorporated into MBGNs, which was probably due to the antioxidant property of Ce [19,62]. It should be pointed out that the cellular uptake of BGNs usually occurs when these nanoparticles are cultured directly with cells [[65], [66], [67]]. Tsigkou et al. [65] investigated interactions between spherical BGNs (215 ​± ​20 ​nm) and stem cells (bone marrow and adipose tissue-derived). They found that a large number of particles could be internalized by the cells but exhibited insignificant effects on cellular performance. Li et al. [67] revealed that the size of MBGNs could affect the internalization and intracellular localization of particles. Based on the knowledge obtained in the literature, we expected that Ce-MBGNs could be internalized by macrophages. However, a comprehensive investigation is still required to understand the internalization of Ce-MBGNs and their consequent effects on cellular responses.

The antioxidant activity of Ce-BGs has been evaluated under acellular conditions [22,23]; however, its influence on macrophages has not been widely investigated. We thus evaluated the expression of oxidative stress related–genes in macrophages cultured with Ce-MBGNs. In comparison with the MBGN group and the control, the expression of NOS2, a gene related to the production of nitric oxide, was significantly downregulated by Ce-MBGN groups (Fig. 8), which is in line with the previously reported results [19]. The downregulation of NOS2 expression was even more prominent in the presence of the oxidizing agent NPS, indicating the significant antioxidant effect of Ce-MBGNs. These results are consistent with those obtained in the inflammatory assay, in which MBGNs were found to show proinflammatory responses while Ce-MBGNs inhibited the expression of proinflammatory genes (Fig. 7). This behavior could be ascribed to the changes between oxidation states (Ce^4+^ and Ce^3+^) allowing to scavenge ROS and reactive nitrogen species ​[44] that are responsible for inflammatory responses [19,62]. Ce usually exists in two oxidation states in melt-derived BGs [23,68], which contain a higher concentration of Ce^3+^ than Ce^4+^ on the surfaces. Similarly, Ce in Ce-MBGNs also exhibited both oxidation states, but a higher concentration of Ce^4+^ on the surface was observed (Fig. 4). The presence of Ce^3+^/Ce^4+^ redox couple results in a unique redox chemistry that can further modulate a series of biological responses (e.g., suppression of oxidative stress, osteogenesis, and angiogenesis) [44,69]. The coexistence and the surface ratio of Ce^3+^/Ce^4+^ in Ce-containing materials are thus considered to play a key role in the enzyme mimetic, antioxidant, and osteogenic activities [44]. Overall, the results presented suggested that the antioxidant effect of Ce-MBGNs could protect cells from oxidative stress and consequently inhibit inflammatory responses to some extent.

Bone regeneration involves multiple biological responses including early inflammation, angiogenesis, and osteogenesis [70]. *In vitro* and *in vivo* osteogenic effects of nanoceria through modulating oxidative stress and inflammatory response have been evidenced [18,71]. In comparison with MBGNs, Ce-MBGNs could significantly downregulate the expression of MMP9 in macrophages (Fig. 8), probably related to their effect on the downregulation of IL-1β (Fig. 7) [72], which could thus potentially modulate bone remodeling and regeneration [73]. In comparsion with MBGNs (Fig. 7), Ce-MBGNs significantly reduced the expression of the proinflammatory genes L-1β, IL-6, and TNF-α. The expression of IL-1β and IL-6 genes was even downregulated by 0.2 M Ce-MBGN in comparison with the control. In inflammatory bone diseases, elevated systemic levels of TNF can stimulate the generation of osteoclast precursors (OCPs) in the bone marrow and can also enhance their egress into the bloodstream, through which inflammatory responses can be maintained and even amplified [64,74]. OCPs can differentiate into osteoclasts and increase the production of many factors in response to TNF and RANKL, which in turn increase the number of OCPs and negatively affect bone volume and turnover [75]. Ce-MBGNs could thus be ​mediators to inhibit pro-osteoclastogenic responses toward ​enhanced osteogenesis [17,76]. They are therefore attractive for the treating of diseases affecting bone tissue repair and remodeling under inflammatory conditions, such as osteoarthritis and osteoporosis [73,77].

**Fig. 10:**
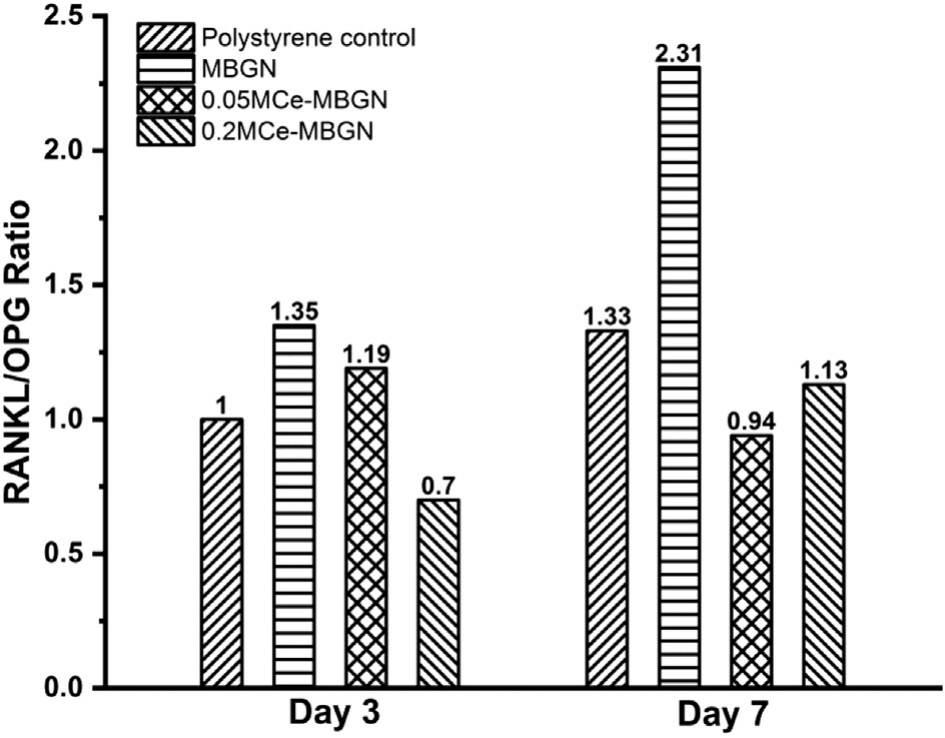
Calculated RANKL/OPG ratio of osteoblast-like SAOS-2 ​cells cultured with MBGN and Ce-MBGN on day 3 and day 7. RANKL/OPG, receptor activator of nuclear factor kappa-Β ligand/osteoprotegerin; MBGN, mesoporous bioactive glass nanoparticles; Ce-MBGN, cerium-containing MBGN.

Ce-MBGNs can also regulate the formation of multinucleated osteoclasts from their precursors and their activation and survival by modulating the RANKL/RANK signaling pathway [78]. It is known that OPG protects skeletons from excessive bone resorption by binding to RANKL and prevents RANKL from binding to its receptor RANK [79,80]. Thus, the RANKL/OPG ratio is an important indicator of osteogenesis [81], which was calculated for all the groups (Fig. 10). The MBGN group showed a higher RANKL/OPG ratio than the control at both time points, which indicated that the ratio was unbalanced toward ​bone resorption. On the other hand, 0.2 M Ce-MBGN showed a higher expression of OPG than RANKL on both day 3 and day 7, with a RANKL/OPG ratio unbalanced toward ​OPG in comparison with the control and MBGN group, suggesting a higher pro-osteogenic effect [81]. An overexpression of ALPL was induced by the osteoblast-like SAOS-2 ​cells in contact with MBGNs on day 7, which was in agreement with previous results [65,82]. As a comparison, the presence of Ce in MBGNs seemed to inhibit the expression of ALPL, which could be related to their effects in the downregulation of TNFα expression, as TNFα could stimulate ALP activity and mineralization [83].

These preliminary *in vitro* biological assessments evidenced the biocompatibility of Ce-MBGNs and their anti-inflammatory responses. In addition, the pro-osteogenic potential of Ce-MBGNs was also preliminarily evidenced. Ce-MBGNs can thus potentially modulate bone remodeling and regeneration by tuning inflammatory responses of relevant cells [18,84,85] and also have the potential to reduce inflammatory responses provoked on the tissues surrounding the implanted material [19].

## Conclusions

We successfully synthesized Ce-MBGNs by combining a microemulsion-assisted sol-gel method and a postmodification approach. Careful control of the postmodification parameters (e.g., temperature, solution concentration) allowed the modified nanoparticles to preserve the high dispersity, particle shape/size, and internal mesoporous structure. The formation of nanoceria clusters could be avoided during the postsurface modification, which ensured the homogeneous chemical composition of Ce-MBGNs. The incorporated Ce exhibited both Ce^3+^ and Ce^4+^ oxidation states, which induced their antioxidant activity. Importantly, in comparison with MBGNs and polystyrene culture plate control, Ce-MBGNs exhibited anti-inflammatory responses in culture with macrophages and pro-osteogenic activity in culture with osteoblast-like cells. The synthesized Ce-MBGNs showed great potential as building blocks for a variety of advanced biomedical devices, particularly to target inflammatory bone diseases (e.g., osteoporosis) and infected bone defects, considering their antioxidant, anti-inflammatory, and pro-osteogenic activities.

## Author contributions

K.Z. contributed to conceptualization, methodology, validation, investigation, writing the original draft, reviewing and editing and supervision. E.T. contributed to methodology, validation, investigation and writing the original draft. A.B. contributed to validation and investigation. N.T. contributed to validation and investigation. C.C. contributed to validation and supervision. M.M. contributed to validation and supervision. S.F. contributed to validation, supervision ​and writing, reviewing and editing. C.V.-B. contributed to validation, supervision, project administration and funding acquisition. G.I. contributed to methodology, validation, writing, reviewing and editing and supervision. A.R.B. contributed to conceptualization, methodology, supervision, writing, reviewing and editing, project administration and funding acquisition.

## Conflict of interest statement

The authors declare that they have no known competing financial interests or personal relationships that could have appeared to influence the work reported in this paper.
