# Supplementary material for: Antioxidant mesoporous Ce-doped bioactive glass nanoparticles with anti-inflammatory and pro-osteogenic activities
Source: Mater Today Bio. 2020 Jan 9;5:100041. doi: 10.1016/j.mtbio.2020.100041 (PMC7083763; doi:10.1016/j.mtbio.2020.100041)
Supplement: Multimedia component 1 [file mmc1.docx]

**Supporting Information**

**Antioxidant mesoporous Ce-doped bioactive glass nanoparticles with anti-inflammatory and pro-osteogenic activities**

Kai Zheng^1*^, Elisa Torre^2^, Alessandra Bari^3^, Nicola Taccardi^4^, Clara Cassinelli^2^, Marco Morra^2^, Sonia Fiorilli^3^, Chiara Vitale-Brovarone^3^, Giorgio Iviglia^2^, Aldo R. Boccaccini^1*^

^1^Institute of Biomaterials, University of Erlangen-Nuremberg, Erlangen, Germany

^2^Nobil Bio Ricerche Srl, Portacomaro d’Asti, Italy

^3^Department of Applied Science and Technology, Politecnico di Torino, Turin, Italy

^4^Institute of Chemical Reaction Engineering, University of Erlangen-Nuremberg, Erlangen, Germany

*Corresponding authors:

E-mail address: [kai.zheng@fau.de](mailto:kai.zheng@fau.de) (K. Z.); aldo.boccaccini@fau.de (A. R. B.)


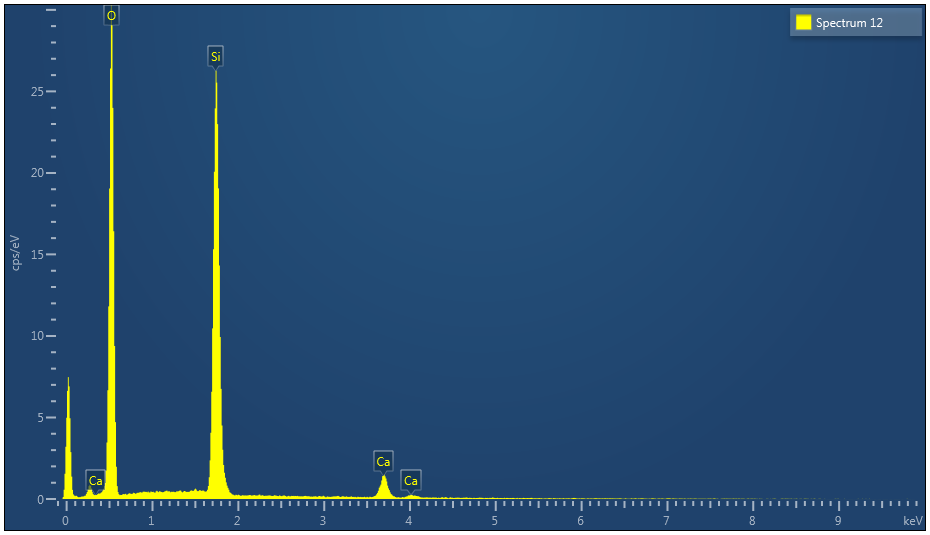

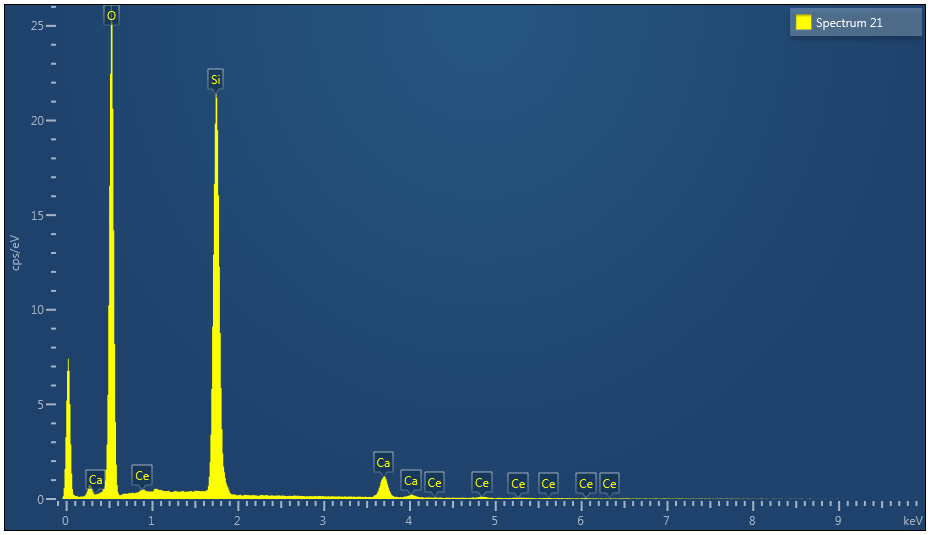

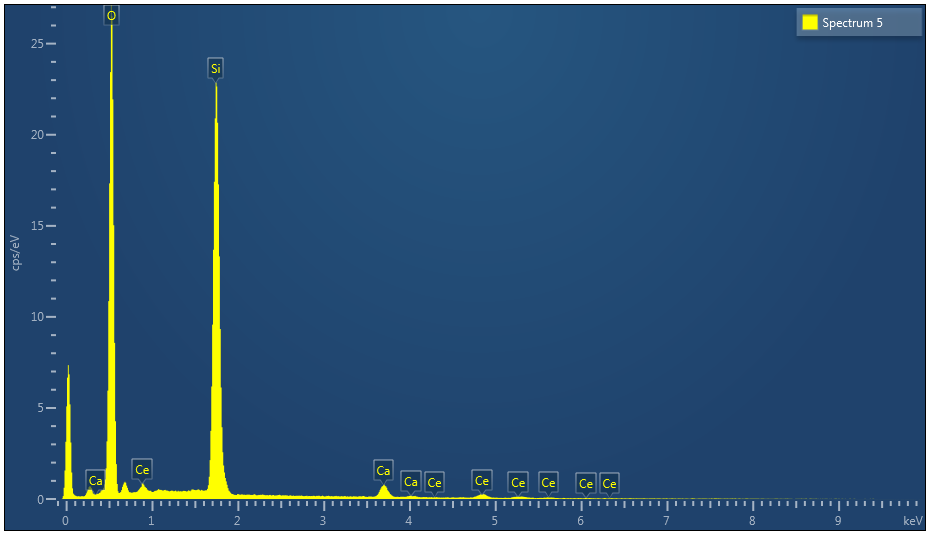


**1.3 mol% of Ce**

**2.2 mol% of Ce**

**0.2MCe-MBGN**

**0.05MCe-MBGN**

**MBGN**

**Fig. S1** EDS spectra of MBGN, 0.05MCe and 0.2MCe-MBGN. Inserted the concentration of incorporated Ce that was calculated using the atomic ratio in the EDS results.

**60^o^C, 0.2M**

**80^o^C, 0.2M**




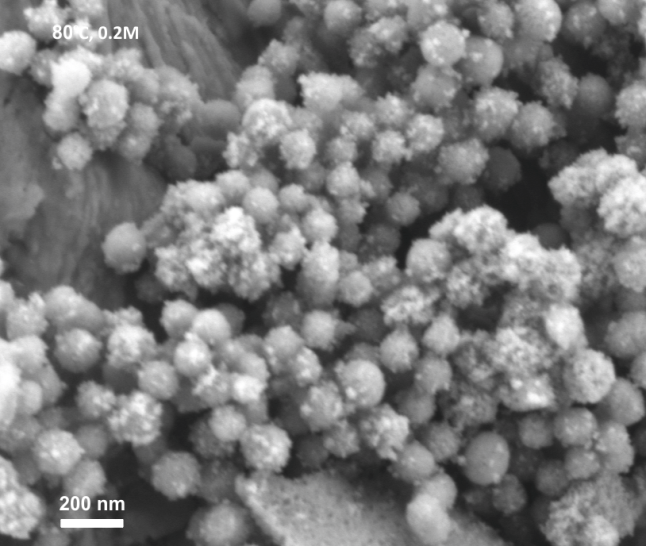

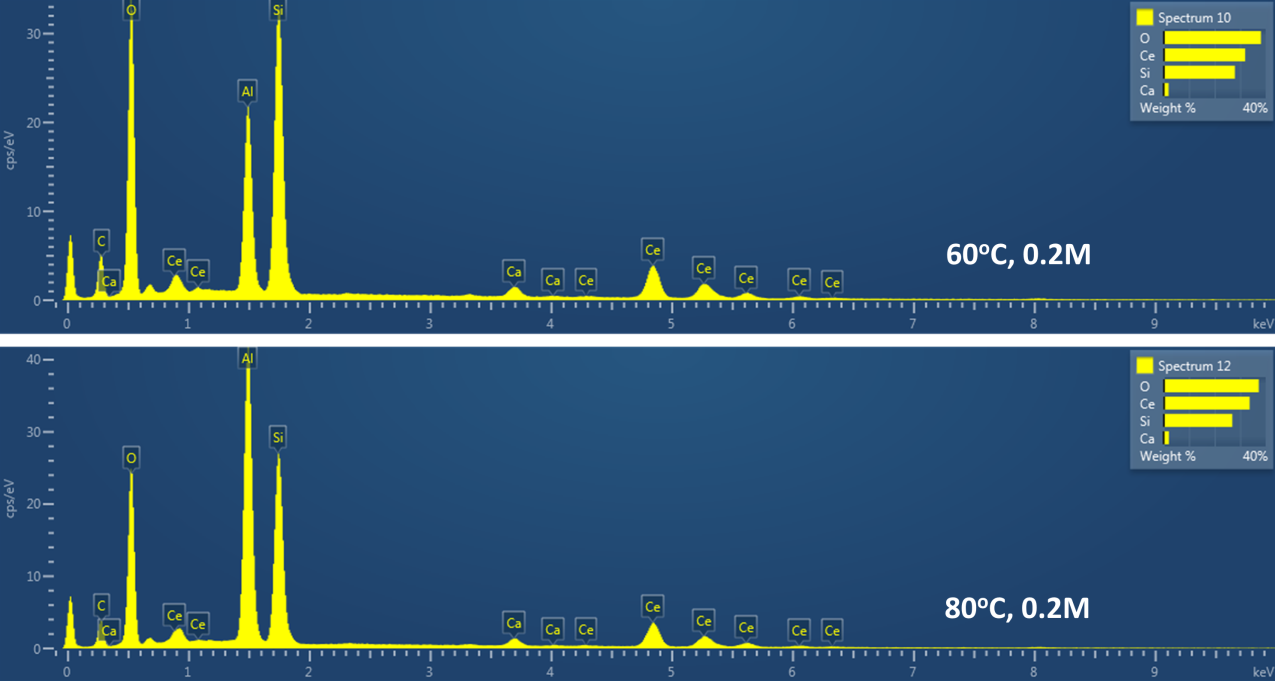


**(b)**

**(a)**

**18 mol% of Ce**

**13 mol% of Ce**





**(C)**

**JCPDS No: 34-0394 of CeO_2_ crystal.**

**Fig. S2** (a) SEM images and (b) EDS spectra of Ce-MBGN modified at 60^o^C and 80^o^C in 0.2M cerium nitrate ethanol solution indicating the formation of nanoceria. Inserted the concentration of incorporated Ce that was calculated using the atomic ratio in the EDS results. (c) XRD patterns of Ce-MBGN modified at 60^o^C and 80^o^C prove the formation of nanoceria.


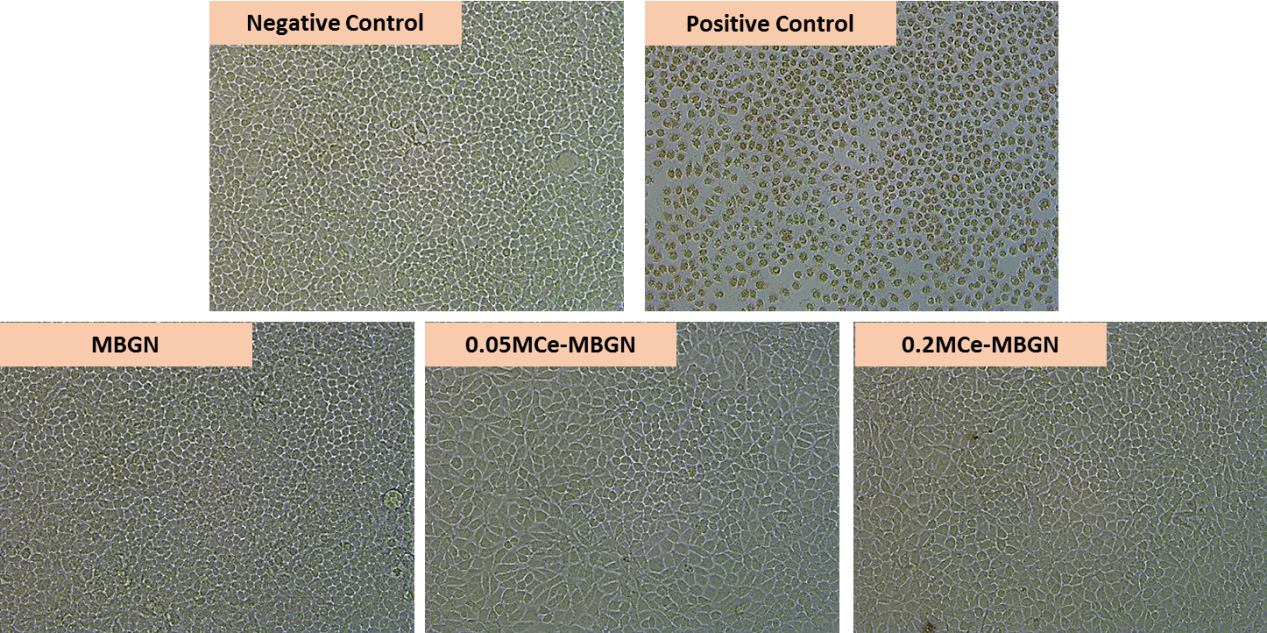


**Fig. S3** Optical images of fibroblast cells cultured in direct contact with MBGN and Ce-containing MBGNs at the concentration of 1 mg/mL.
